# Development and Characterization of Xanthan Gum and Alginate Based Bioadhesive Film for Pycnogenol Topical Use in Wound Treatment

**DOI:** 10.3390/pharmaceutics13030324

**Published:** 2021-03-03

**Authors:** Cinzia Pagano, Debora Puglia, Francesca Luzi, Alessandro Di Michele, Stefania Scuota, Sara Primavilla, Maria Rachele Ceccarini, Tommaso Beccari, César Antonio Viseras Iborra, Daniele Ramella, Maurizio Ricci, Luana Perioli

**Affiliations:** 1 Department of Pharmaceutical Sciences, University of Perugia, 06123 Perugia, Italy; mariarachele.ceccarini@unipg.it (M.R.C.); tommaso.beccari@unipg.it (T.B.); maurizio.ricci@unipg.it (M.R.); 2 Civil and Environmental Engineering Department, University of Perugia, 05100 Terni, Italy; debora.puglia@unipg.it (D.P.); francesca.luzi@unipg.it (F.L.); 3 Department of Physics and Geology, University of Perugia, 06123 Perugia, Italy; alessandro.dimichele@collaboratori.unipg.it; 4 Istituto Zooprofilattico dell’Umbria e delle Marche, 06126 Perugia, Italy; s.scuota@izsum.it (S.S.); s.primavilla@izsum.it (S.P.); 5 Department of Pharmacy and Pharmaceutical Technology, Faculty of Pharmacy, University of Granada, Campus of Cartuja, 18071 Granada, Spain; cviseras@ugr.es; 6 Department of Chemistry, College of Science and Technology, Temple University, Philadelphia, PA 19122, USA; daniele.ramella@temple.edu

**Keywords:** pycnogenol, xanthan gum, sodium alginate, hydrogel film, bioadhesion, wounds

## Abstract

Pycnogenol (PYC) is a concentrate of phenolic compounds derived from French maritime pine; its biological activity as antioxidant, anti-inflammatory and antibacterial suggests its use in the treatment of open wounds. A bioadhesive film, loaded with PYC, was prepared by casting, starting with a combination of two biopolymer acqueous solutions: xanthan gum (1% wt/wt) and sodium alginate (1.5% wt/wt), in a 2.5/7.5 (wt/wt) ratio. In both solutions, glycerol (10% wt/wt) was added as plasticizing agent. The film resulted in an adhesive capable to absorb a simulated wound fluid (~ 65% wt/wt within 1 h), therefore suitable for exuding wounds. The mechanical characterization showed that the film is deformable (elastic modulus E = 3.070 ± 0.044 MPa), suggesting adaptability to any type of surface and resistance to mechanical solicitations. PYC is released within 24 h by a sustained mechanism, achieving a maximum concentration of ~0.2 mg/mL, that is safe for keratinocytes, as shown by cytotoxicity studies. A concentration of 0.015 mg/mL is reached in the first 5 min after application, at which point PYC stimulates keratinocyte growth. These preliminary results suggest the use of PYC in formulations designed for topical use.

## 1. Introduction

Pycnogenol (PYC) is the registered trade name of a special standardized extract obtained from the bark of the French maritime pine, *Pinus pinaster ssp.*, species Atlantica, family Pinaceae, genus Pinus. It is grown in large monocultures, especially in the South-western French area of Biscay [1]. 

PYC is a concentrate of phenolic compounds (phenolic acids, catechin, epicatechin, taxifolin and procyanidins), present in both the free and the glycosylated forms [1]. 

These molecules are responsible for PYC’s biological activity, as it has been known since the ancient era. It was mentioned by Hippocrates as a remedy for inflammatory diseases, and in the Thesaurus Medicaminum (1479) as a wound healing adjuvant [2].

The pharmacological activity of PYC has been reported in several studies, during which both radical-scavenging and anti-inflammatory properties were observed [1,3,4,5,6,7]. 

The flavonoids which can prevent free radicals from forming resonance-stabilized phenoxyl radicals are responsible for PYC’s antioxidant properties [8]. The anti-inflammatory activity can instead be ascribed to PYC’s ability to up-regulate the expression of gene coding for 5-lipoxygenase and cyclooxygenase-2, as well as inhibit phospholipase A2 [9].

Moreover, PYC’s antibacterial activity toward gram-positive (as *E. faecalis*, *Clostridium perfringens, S. aureus*) and gram-negative (as *E. coli*, *K. pneumoniae*, *P. aeruginosa*) bacteria was observed [10].

Recent studies highlighted that PYC can promote the synthesis of molecules present in the extracellular matrix such as hyaluronic acid and collagen [6].

The combination of the antioxidant, anti-inflammatory and antibacterial activities combined with the stimulation of extracellular matrix regeneration, makes PYC an interesting product for use in wound treatment formulations.

Several studies about the presence of PYC in topical products, such gels and creams intended for wound application are present in literature [7,11]. However, such formulations show a limited residence time and are not able to protect the damaged area.

For this reason, the use of advanced formulations is necessary to perform a prompt wound treatment, preventing bacteria invasion of the damaged skin and severe inflammation factors responsible for delayed healing. With these aspects in mind, a suitable formulation for wound treatment should: (i) contain an active ingredient able to promote the repair process, (ii) cover the wound protecting it from mechanical damage and bacterial invasion, and (iii) remove the excess exudate. Recent studies report in-situ gel forming systems loaded with PYC [12] as viable alternatives.

The purpose of this study was to develop an effective biocompatible formulation that would be safe for the patient and environmentally friendly. Thus, films were realized using two biopolymers: xanthan gum and sodium alginate, FDA approved as G.R.A.S. (generally recognized as safe) [13,14,15,16]. The study was divided in three steps: (i) identification of the best film composition and preparation method, (ii) investigation of unloaded film characteristics, and (iii) PYC loading and study of the loaded film performances.

## 2. Materials and Methods

### 2.1. Materials

Xanthan gum was purchased by Multiagency S.n.c. (Cava Manara, PV, Italy). Alginic acid sodium salt, calcium chloride dihydrate were supplied by Sigma Aldrich (Milano, Italy). Pycnogenol (PYC) dry extract tit. French maritime pine 65% OPCS was supplied by A.C.E.F. s.p.a, Fiorenzuola d’Arda (Piacenza, Italy). Magnesium chloride was purchased from Carlo Erba Reagents S.r.l. (Milano, Italy). Ultrapure water was obtained by reverse osmosis process in a MilliQ system Millipore (Roma, Italy). Other reagents and solvents were of analytical grade and used without further purification. The pH 6.5 simulated wound fluid (SWF) was prepared by dissolving 8.30 g of NaCl and 0.28 g of CaCl_2_ in 1000 mL of ultrapure water [17].

### 2.2. Methods

#### 2.2.1. Film P reparation

Films were prepared by solvent casting method [17] starting from binary mixtures of biopolymer-based hydrogels of alginic acid sodium salt (AL) and xanthan gum (XG) glycerol (10% wt) used as plasticizing agent for the final films. The AL based hydrogel was prepared under magnetic stirring (600 rpm) by dispersing the biopolymer in the water previously added by glycerol. XG based hydrogel was prepared using mortar and pestle. XG was previously wetted with glycerol and then hydrated with bidistilled water.

As far as loaded films are concerned, AL and XG, 5% wt/wt of PYC, hydrogels were solubilized in the bidistilled water later used for hydrogel preparation [11].

Film prototypes were obtained using binary mixtures of (wt/wt) of AL/XG hydrogels in different ratios. To remove the air incorporated during the mixing, AL and XG hydrogel and the corresponding blends were degassed by an ARE-250 mixer (THINKY, Kidlington, England) at 2000 rpm for 3 min (mixing) and at 2000 rpm for 5 min (defoaming), at room temperature (RT). The hydrogel mixture (56.0 g) was casted into circular Teflon moulds (diameter 14 cm) and placed in the oven at 37.0 °C ± 0.1 for 24 h. Afterwards, the films were treated with of a 5% (wt/v) solution of CaCl_2_·2 H_2_O (6.0 mL) and placed again in the oven at 37.0 °C ± 0.1 for further 24 h. After this time, the dried films were removed from the mould and stored under CaCl_2_.

#### 2.2.2. Film Storage Conditions

Three storage conditions were evaluated to optimize the preservation of the films’ original properties:

(1) CaCl_2_ (relative humidity, R.H. 40%) at R.T.,
(2) saturated MgCl_2_ solution at RT (R.H. 33%),
(3) saturated MgCl_2_ solution at 4.0 °C (R.H. 34%).

#### 2.2.3. Thermogravimetric Analyses

Thermogravimetric measurements of raw materials and films were performed by using an Exstar 6300 TG/DTA system (Seiko, Woodland, CA, USA). Each film was cut in similar portions (weight 10 mg) and placed inside small alumina crucibles, under controlled and inert (nitrogen flow, 200 mL/min) atmosphere. The residual mass of all the films after 1 week of storage in CaCl_2_ desiccators was measured at both 100 °C and 600 °C.

#### 2.2.4. Mechanic Characterization

The tensile tests were performed by a digital microprocessor instrument LLlyod LR30K (Hampshire, USA) The films were cut in portions of 100 mm × 10 mm (UNI ISO 527) to prepare samples with a useful length of 50 mm. The experiments were performed at 5 mm/min, cell load 50 N. Values for maximum stress, deformation at break and elastic modulus were registered. The reported results are an average of five measurements (n = 5). The samples were placed in desiccator containing a saturated MgCl_2_ solution for 1 week at RT until reaching constant weight.

#### 2.2.5. Morphology and Thickness

Film morphology and thickness were evaluated by FE-SEM LEO 1525 ZEISS (Carl Zeiss Microscopy, Jena, Germany). The samples were prepared by deposition of the sample on conductive carbon adhesive tape and then metalized with chromium (8 nm) by sputtering.

#### 2.2.6. Water Content

To measure the water content, each film was cut in squares of 4 cm^2^ and dried, and the weight loss was calculated. Each portion was placed in three different conditions:

(1) ventilated oven at 42 °C,
(2) desiccator under CaCl_2_,
(3) desiccator under P_2_O_5_.

Each sample was weighted before the experiment (Wi) and at set times (Wf) of storage in the above-described conditions (n = 3, ± SD). The weight % was calculated by using Equation (1):

(1) $$\mathrm{Weight}\;\%=\frac{\mathrm{Wi}-\mathrm{Wf}}{\mathrm{Wi}\;}\;\times \;100$$

where W_i_ is the initial weight of the film and W_f_ is the weight after storage.

#### 2.2.7. Water Holding Studies

Film ability to absorb exudates was evaluated by means of hydration percentage (%) and matrix erosion (DS) calculated by Equations (2) and (3), respectively:

(2) $$\mathrm{Hydration}\;\%\;=\;\frac{\;W2-\;W1}{W2}\times \;100$$

(3) $$\mathrm{DS}=\;\frac{W1-W3}{W1}\;\times \;100$$

Each film was cut in portions of 4 cm^2^ (2 cm × 2 cm) and a single portion was weighted (W1), immersed in SWF (5 mL) inside a centrifuge tube (50 mL Corning, Torino, Italy) and held at 32.0 ± 0.1°C for established times (1, 2, 3, 4, 5, 6, 24, 48 h). After immersion, the films were wiped using filter paper to remove the excess surface SWF, and weighted (W2). After hydration, the films were dried at 60°C for 24 h, maintained over CaCl_2_ (RH 40%) for 48 h and reweighted (W3) [17].

#### 2.2.8. Ex Vivo Adhesion Studies

Film ability to bind the skin was evaluated ex vivo using samples (shoulder region) obtained from pigs (Large White, weight ∼ 165–175 kg, furnished by Veterinary Service of ASL N. 1 Città di Castello, Perugia, Italy). The skin samples were used for the assays within 12 h from pig death [18]. The film was attached on a support using cyanoacrylate glue and connected to the dynamometer Didatronic (Whatman GmbH, Dassel, Germany). A piece of porcine skin tissue was fixed with cyanoacrylate glue on the surface of a glass support placed in a thermostatic bath at 32.0 ± 0.5°C. Every film was cut in portions of 2 cm × 2 cm. The free side of the skin was wetted with 50 μL of SWF and put in contact with the film sample by applying a light force for 1 min. The force and time necessary for detachment of the film from the skin was measured and expressed as the average of three measurements (n = 3).

#### 2.2.9. Release Studies

The dissolution tests for transdermal patches using the extraction cell (depth of 2.6 mm, diameter 27 mm, release surface exposed 3.14 cm^2^) prescribed by the European Pharmacopoeia (Ph. Eur. 10^th^ Ed.) was used to evaluate PYC release from the film. The test was performed for 24 h working at 40 rpm in sink conditions using SWF as dissolution medium (400 mL) kept at 32.0 ± 0.5°C. At preset intervals, samples (2 mL) were extracted, replaced by an equal amount of SWF and analyzed by UV–Vis spectrophometer Agilent 8453 (Agilent Technologies, Germany) using a calibration curve in SWF (λ_max_ = 281.0 nm; r^2^ = 0.99) [17].

#### 2.2.10. Antimicrobial Activity

The antimicrobial activity of PYC solution and PYC loaded films was evaluated by a properly adapted agar diffusion method [17]. The assay was performed on the strains reported in Table S1.

Each strain (lyophilized) was suspended in 1.0 mL of sterile demineralized water and then sown in sheep’s blood agar (CM 0271: proteose peptone 15.0 g/L, liver digest 2.5 g/L, yeast extract 5.0 g/L, sodium chloride 5.0 g/L, agar 12.0 g/L, sterile sheep blood 50.0 mL/L, pH 7.4 ± 0.2 at 25.0 °C, OXOID, Thermo Fisher, Ferentino, Italy) to obtain isolated colonies, that were afterwards incubated in conditions specific to each strain (Table S1). After that, a broth culture in BHI (beef heart infusion solids 17.5 g/L, proteose peptone 10.0 g/L, glucose 2.0 g/L, sodium chloride 5.0 g/L di-sodium phosphate 2.5 g/L, pH 7.4 ± 0.2 at 25.0 °C) was prepared from the colony of each strain and incubated overnight at 37.0 ± 0.1 °C. The microorganisms were then counted to determine the optimal dilution for the experiment. The culture medium employed for evaluation of the antibacterial activity (meat extract 3.0 g/L, meat peptone 5.0 g/L, glucose 4.0 g/L, sodium chloride 10.0 g/L, di-potassium phosphate 1.0 g/L, agar noble 13.0 g/L, pH 7.2 ± 0.2 at 25 °C) was dissolved at 100 °C, cooled to 44–47 °C and inseminated with 1.0 mL of bacterial suspension to obtain a final concentration of 10^5^ cells/mL. This suspension was accurately mixed and poured (25 mL) into Petri dishes (90 mm diameter), let it cool on a horizontal surface. At the time of use, PYC was diluted with sterile demineralized water to obtain five concentrations: 10, 1, 0.1, 0.05 and 0.025 mg/mL.

For loaded films, the experiment was performed as follows: a small square (1 cm × 1 cm) of the two films was placed in each series of plates, similar to how the active ingredient free corresponding films were tested. Three agar plates, uninoculated, were incubated to verify medium sterility. The plates incubated in the conditions reported in Table S1, were then measured for inhibition halos by a gauge [17].

#### 2.2.11. Cytotoxicity Assay

The HaCaT cell line (300493, CLS Cell Lines Service, purchased from I.Z.S.l.E.R. (Istituto Zooprofilattico Sperimentale della Lombardia e dell’Emilia Romagna, Italy)) was used as a representative model to appreciate the epidermal homeostasis and healing during wound treatment. HaCaT, a monolayer human immortalized keratinocyte, was purchased from I.Z.S.I.E.R at 46° passage level. The cellular viability was assessed using MTT assay after 24 h of treatment [19]. HaCaT cells was used always between 48°–55° passage and each experiment was performed in triplicate for two times. For MTT assay a 96-well plate was seeded. The final cell density was 1 × 10^4^ cells/well. After 24 h, when the cells reached the 60% of confluence, fresh complete medium was replaced for treatment with PYC samples dilutions from the stock solution prepared incubating the film (1 × 1 cm) with DMEM (10 mL) for 24 h.

MTT reagent (0.5 mg/mL in PBS) was added in each well at 0.05 µg/µl final concentration for 3 h. Then, the supernatant was carefully removed, and the OD values were measured spectrophotometrically (Eliza MAT 2000, DRG Instruments GmbH, Marburg, Germany) and cell viability was expressed as a percentage relative, as previously described [20].

#### 2.2.12. In vitro Wound Healing Assay

CytoSelect™ Wound Healing Assay Kit (Cell Biolabs, Inc., San Diego, CA, USA) was purchased to investigate the effect of PYC released from the film on wound closure in vitro. A 24-weels tissue culture plate containing properly treated inserts was used.

HaCaT cells for these experiments was seeded in DMEM complete medium at the final concentration of 5 × 10^4^/500 μL (1 × 10^5^/mL). After 24 h, when keratinocyte reached 80% of confluence, the inserts were removed from the wells leaving the wound field [21].

The cells were treated with the two different concentrations of PYC obtained incubating the film (1 × 1 cm) with DMEM (10 mL) for 24 h (0.015 mg/mL and 0.030 mg/mL), for 24 further hours [22].

Migration into the wound field was determined as previously described and pictures of control cells (CTR) and treated cells (PYC) after 6, 12 and 24 h were taken and three fields for each condition were compared [22].

The total wound field surface area was calculated considering the dimensions of the insert: Total Surface Area = 0.9 mm (length) × 1.8 mm = 1.62 mm^2^. To measure the % closure, the migration cell surface area was determined for each experiment (Migration Cell Surface = length of cell migration × 2 × 1.8 mm).

The percent closure of wound field was calculated considering three different times of treatment: 6, 12 and 24 h and using Equation (4):

(4) $$\%\;\mathrm{closure}\;=\;\frac{\;\mathrm{migration}\;\mathrm{cell}\;\mathrm{surface}}{\mathrm{total}\;\mathrm{surface}\;\mathrm{area}}\;\times \;100$$

#### 2.2.13. Statistical Analysis

Results were reported as mean ± standard deviation (mean ± SD). One-way ANOVA test was used for statistical analysis. Differences were considered statistically significant for *p* < 0.05.

## 3. Results and Discussions

Bioadhesive films are useful to overcome problems commonly reported about conventional wound dressings. One of the main limitations is the use of adhesives to promote the adhesion to skin resulting in a painful and traumatic removal with consequent damage of the surrounding tissue. The development of bioadhesive medications, based on biopolymers and thus easily removable by washing, could be a suitable alternative. With this goal in mind, the focus was shifted to the use of natural biopolymers, approved by FDA and EMA and used in products for health care and thus safe for use (classified as G.R.A.S.).

Films based on the use of biopolymers could allow a suitable wound treatment due to properties such as compatibility with tissues, high ability to hold water, and to provide a moist environment protecting the wound from desiccation, infections and mechanical solicitations [23].

The biopolymers for the development of the bioadhesive films were selected according to the following set requirements: (i) adhesion capacity to skin surface [24], (ii) high residence time in the application site, (iii) easy and atraumatic removal (e.g., by washing), (iv) sustained release of the active ingredient, (v) mechanical protection of the damaged area [25], and (vi) eco-friendly. Xanthan gum (XG) was found to be a viable material; it displays good water-solubility and excellent biocompatibility, and it is non-toxic and not irritant to the skin. It was therefore chosen as the optimal polymer for film preparation. Initially, hydrogels based on XG (0.5%, 1% and 2% wt/wt) were prepared and used in film formation. However, unsatisfying results were obtained and its mixing with another natural biopolymer was devised. Sodium alginate (AL) was selected because of its use as gelling, thickening and film forming agent [26].

### 3.1. Unloaded Film Preparation and Characterization

After preliminary studies based on the evaluation of hydrogels characteristics (homogeneity and consistency), easy of casting and final film appearance (imperfection detected by visual inspection), the most suitable compositions of the starting hydrogels used to prepare the films were the follows: hydrogel-AL: AL 1.5% (wt/wt), glycerol 10% (wt/wt), bidistilled water until 100 g; hydrogel-XG: XG 1% (wt/wt), glycerol 10% (wt/wt), bidistilled water until 100 g. Glycerol was chosen as plasticizing agent as observed in other studies [17,27].

Different ratios of hydrogel-AL/hydrogel-XG (5.0/5.0; 1.5/8.5; 8.5/1.5; 7.5/2.5; 2.5/7.5; 2.0/8.0; 1.0/9.0 wt/wt) were considered and a preliminary evaluation based on both easy production (bubbles removal and casting) and film final properties (adhesion to skin, flexibility, resistance to traction, application, and removal by washing) was performed. In the end, the hydrogels showing the best compositions were A and B (Table 1); the corresponding films (Film A and Film B) were thus produced and fully characterized.

### 3.2. Storage Conditions

Storage conditions represent a critical point for films; modifications of water content during the shelf life could decrease it performance during the application phase. Inadequate temperature and humidity conditions could be responsible for softening and/or stiffening. With this in mind, the prepared films were removed from the mould and placed in desiccators under a diverse range of storage conditions (Table S2) and submitted to visual inspection and water content determination after 7 days. All the indications deriving from this assay can therefore be useful for planning the suitable packaging of the formulation.

Storage in a close system (desiccator) was investigated using CaCl_2_ and MgCl_2_ as they are the most commonly used desiccants [28,29]. Films stored under saturated MgCl_2_ solution at 4 °C appeared gelatinous and therefore difficult to handle and sticky in comparison to a fresh film; this was probably due to rehydration and gelation-type effects on alginate. The storage conditions under CaCl_2_ and under saturated MgCl_2_ solution at RT resulted as the most suitable ones, without significant modifications of the films during storage. Storage under CaCl_2_ was chosen for further studies based on the evaluation of water content in the films.

### 3.3. Water Content Measurement

**Figure 1:**
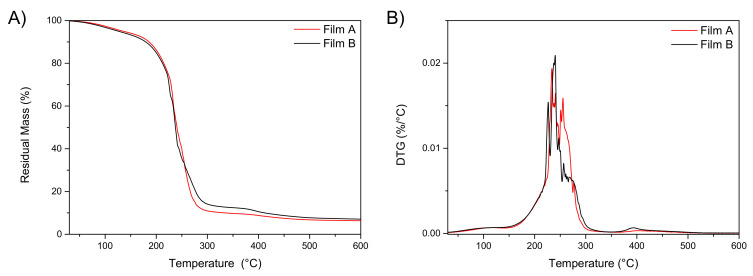
TGA and DTA profiles of film A and film B (*n* = 3).

The evaluation of the residual water content in the films is important, as it influences the performances of the formulation in terms of flexibility, adhesivity and mechanical properties. The amount of water in films A and B after storage under CaCl_2_ was measured by two different approaches. The first approach consisted in the measurement of the water content by dynamic thermogravimetric curves (TGA), while the second estimation was made by means of weight loss calculations after isothermal storage at different constant temperatures in the oven for 24 h. Results of thermogravimetric analysis (TGA) for XG, AL and glycerol as raw materials are reported in Figure S1, while TG and DTG thermograms for the films are included in Figure 1A,B. These experiments were performed to calculate the volatile content of the different materials at low temperatures; specifically, the measurements were done to estimate the residual water content and relate it to film properties [30].

AL thermogram displayed two distinct stages (Figure S1a). The first one, in the range of 30–160 °C, with a maximum decomposition rate at 104.0 °C, is attributable to elimination of water adsorbed by the hydrophilic polymer. The second one, in the range of 210–310 °C with a maximum decomposition rate at 255.7 °C (Figure S2b), was ascribed to a complex process including dehydration of the saccharide rings, depolymerization with the formation of water, CO_2_ and CH_4_. The temperature, at which 50% weight loss happens, was found to be 300 °C for AL [30]. XG thermogram showed single step thermal degradation, following an initial weight loss due to the removal of moisture. The polymeric thermal degradation starts at 200 °C; the main peak is centred at 302.0 °C with a weight loss of around 52% (Figure S1a). The rate of weight loss increases initially, but after 50% weight loss, the rate was found to decrease [31]. The thermogravimetric analysis of glycerol showed single step thermal degradation centred at 255.7 °C (Figure S1a) [31].

In both cases, the residual water content was estimated to be below 4% at 100.0 °C (Figure 1A, residual mass curve). Figure 1B shows the derivative curves (DTG) of neat film A and B, characterized by the presence of a multi-step degradation behaviour. The first peak, centred at 100.0 °C, is attributed to the evaporation of water content. Assumed that the dried XG and AL did not undergo weight losses during heating [32], the measured weight loss below 100.0 °C can be exclusively ascribed to water content; no substantial differences were found for the two differently formulated films. At higher temperatures, the films show one main degradation step at 240–280 °C, attributed to the main fractions in the films composition i.e., glycerol, XG and AL. The third peak at around 400.0 °C is related to the presence of alginate component [23].

The film’s water content was also calculated by measuring the weight modifications under different storage conditions and applying Equation (1). Table 2 shows that storage in the oven at 42 °C allows the highest removal of water after 24 h. On the other hand, the results obtained using CaCl_2_ and P_2_O_5_ are comparable. In all cases, the overall water removal was very low, suggesting that storage in oven at 42 °C is the most efficient method to remove residual water. The residual water content remains high when compared to the results obtained from TGA measurement, performed in dynamic heating conditions.

### 3.4. Mechanical Characterization

The mechanical characterization of films is essential as these formulations were designed to be applied on skin and to conform to every type of surface. For this reason, more information about its elastic response is necessary. The mechanical properties of hydrophilic polymers and of edible matrices are strongly influenced by RH as the humidity acts as plasticizer [33].

Moreover, films must be resistant to mechanical solicitations to which they are subjected (e.g., during removal from packaging, application and period of residence on the skin surface). According to this, evaluation of tensile properties was performed by using a dynamometer.

**Figure 2:**
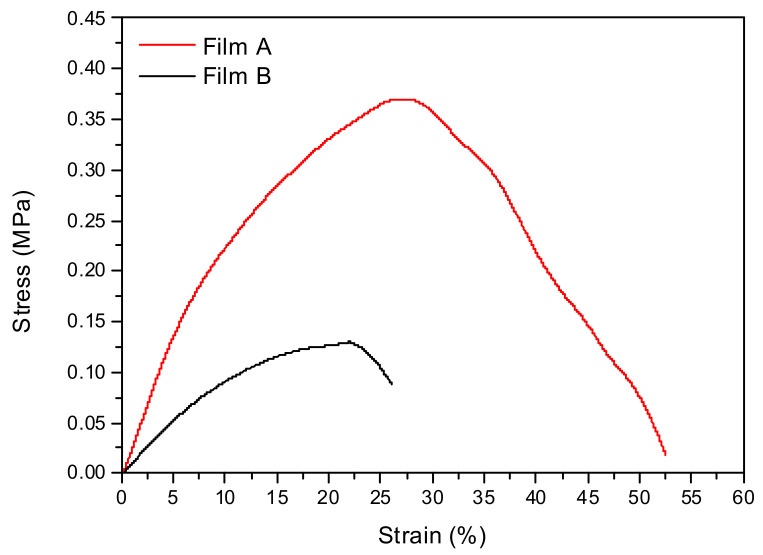
Stress- strain curved of film A (red line) and film B (black line), (*n* = 5).

For each formulation maximum stress (σ_max_), deformation at maximum strength (ε_at σmax_) and elastic modulus (E) were measured (Table 3). The analysis of stress-strain curves of unloaded films (Figure 2) showed that film A is more deformable than film B, as confirmed by the higher value for strain at break, suggesting that the composition of film A could be useful for the fixed objective. We found that a higher quantity of alginate induces an improvement of tensile characteristics, contributing a detectable increase in maximum strength, elastic modulus, and deformation at break [34].

When used without the addition of any plasticizing agent AL and XG show values of deformation lower than 3%; [33,35] the values requested for normal skin are between 61 and 70% [36,37]. This problem can be overcome using glycerol its chemical structure is able to retain water molecules, and storing in controlled-humidity environment. The amount of plasticizer in films A and B is, respectively, 8.9 and 9.3 times greater than the total quantity of the polymeric matrix. Such a high amount of plasticizer greatly influences the mechanical response of the overall systems. As the AL content increases, high values of tensile stress at break are measured (Table 3); this is in agreement with other authors’ studies [33], suggesting that AL is the main responsible for film elasticity and deformability.

### 3.5. Morphology and Thickness

**Figure 3:**
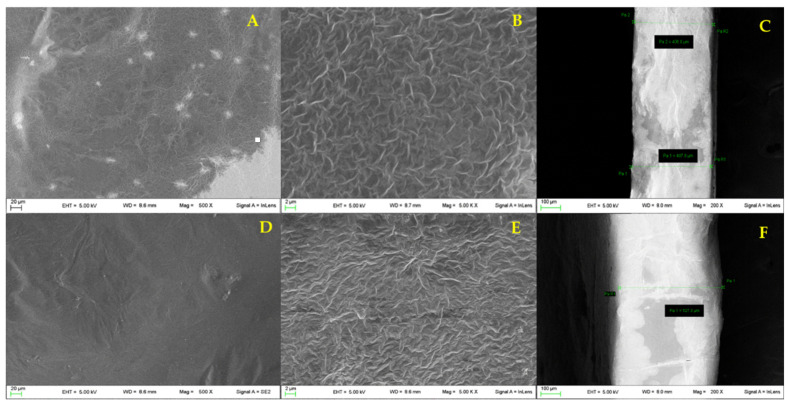
Micrographs of the surface for film A (**A**,**B**) and film B (**D**,**E**); thickness of film A (**C**) and film B (**F**) (*n* = 3).

The morphology and the thickness of both films were studied by scanning electron microscopy (Section 2.2.5). The two films show similar morphology (film A: Figure 3A,B and film B: Figure 3D,E); in particular, a wrinkled surface is detectable. The surface roughness is an important property for bioadhesive systems as it increases the surface area available for adhesion. The films’ thickness was also measured (Figure 3C,F, respectively); film A resulted more compact and thinner (410 ± 2.5 μm) compared to film B (529.4 ± 8.7 μm). This difference is probably attributable to the high AL content in film A, responsible for a more compact and reticulated structure.

### 3.6. Water Holding Studies

**Figure 4:**
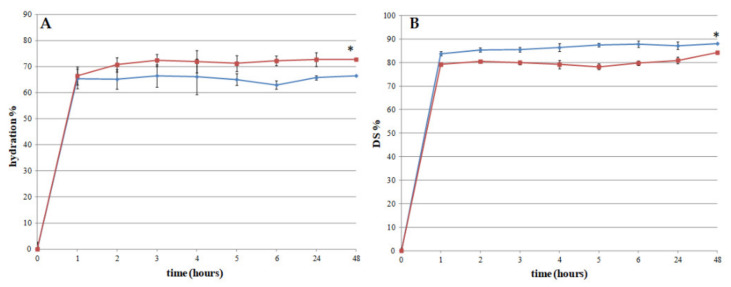
Hydration capacity (**A**) and erosion matrix (**B**) of film A (blue diamonds) and film B (red squares) (*n* = 3); * *p* > 0.05.

The films’ ability to absorb wound exudate was evaluated in vitro by water holding studies. The obtained results (Figure 4A) show that both films hydrate rapidly after contact with SWF. After 1 h, the amount of absorbed SWF is ~ 65% wt/wt for both tested formulations. In the case of film A, this value is maintained until the end of the experiment (48 h), suggesting that the water uptake is immediate and does not change. For film B instead, it increases slightly to 72% after 48 h.

The differences, although minimal, can be ascribed to the different contents of the two biopolymers XG and AL. It is known that XG has better water binding ability than AL [38]. The water molecules bind, mainly by hydrogen bonding, to anionic groups localized in the side chains of XG backbone. Xanthan gum has many groups able to bind water molecules. On the other hand, AL bind water molecules by means of the carboxyl groups [38]. As film A contains lower XG amount than film B (Table 3), the amount of water absorbed by film A is less than that absorbed by film B. Moreover, due to the high AL content, film A could show a high reticulation limiting the number of water molecules entrapped in the polymeric network.

These results suggest that both formulations have suitable swelling capacity, an important prerequisite to obtain an effective release of the active ingredient in the application site.

Another important property required for the developed films is the ease of removal from skin surface. To investigate this aspect, the matrix erosion capacity or dissolution (DS) of each film was evaluated. The obtained results (Figure 4B) show that film A has the higher DS ~ 88% (after 48 h) vs ~ 84% (after 48 h) of film B. The weight loss can be attributed to a loss in the plasticizer agent (glycerol) as well as to XG solubilisation. The profiles obtained from the two films are comparable and the differences are not statistically relevant (*p* > 0.05), suggesting that the different amounts of XG do not significantly modify the behaviour of the two different formulations.

### 3.7. Ex Vivo Adhesion Capacity

The film’s bioadhesion ability was evaluated by ex vivo studies. The force necessary for detachment and the adhesion times were measured for both the unloaded films A and B. The obtained results showed that film A has higher adhesion force (0.20 N ± 0.17 vs 0.07 N ± 0.01) and adhesion time (14.66 ± 5.68 sec vs 6.66 ± 0.57 sec) than B. The adhesion force value measured for film A is attributable to its hydrophobic character likely due to accessibility of the hydrophobic groups of the polymers to skin. In the case of film A, the hydrophilic groups of AL (-OH, -COOH) can bind XG hydrophilic groups (-OH, -COOH). A lower content of AL in film B increases the number of available hydrophilic groups yielding a decreased capacity of adhesion to skin.

### 3.8. Loaded Film Preparation

**Figure 5:**
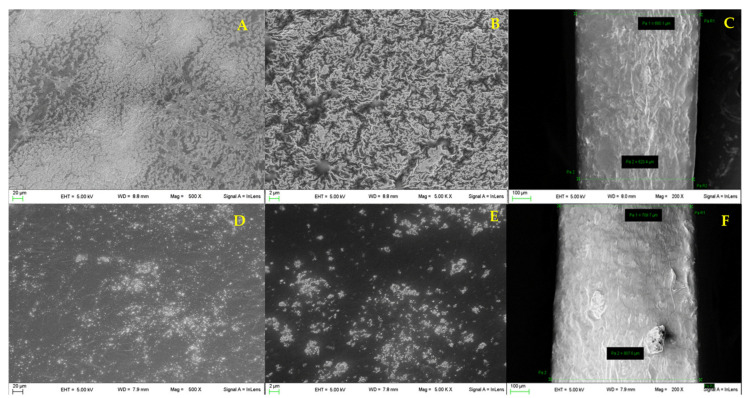
Micrographs of the surface of film A-loaded (**A**,**B**) and film B-loaded (**D**,**E**) and thickness of film A-loaded (**C**) and film B-loaded (**F**) (*n* = 3).

Loaded films A and B were prepared according to the procedure described in the methods section (Section 2.2.1). The amount of PYC in the starting hydrogels was set to 5% wt. according to literature data documenting the high performances in terms of wound healing activity of a semisolid loaded formulation [11]. The following loaded hydrogels were prepared: PYC-hydrogel-AL: AL 1.5% (wt/wt), PYC 5% (wt/wt), glycerol 10 % (wt/wt), bidistilled water until 100 g; PYC-hydrogel-XG: XG 1% (wt/wt), PYC 5% (wt/wt), glycerol 10% (wt/wt). The blends reported in Table 1 were prepared using these two hydrogels. The obtained films, Film A-Loaded and Film B-loaded, were devoid of imperfections and easily removable from the mould, suggesting that PYC presence does not interfere with the film formation. The morphology of the loaded film was evaluated as well (Figure 5). Film A-loaded shows a uniform wrinkled surface (Figure 5A,B), while in film B-loaded (Figure 5D,E), small particles due to PYC inclusion in the polymeric matrix can be detected. The surface analysis of film A-Loaded confirms the presence of a morphology similar to the unloaded one (Figure 3A,B). The thickness of the loaded films, reported in Figure 5C (film A-loaded) and 5F (film B-loaded), resulted increased 1006 ± 31 µm vs 408.8 ± 10.2 µm (film A-loaded and film A respectively), 560.0 ± 29.0 µm vs. 529.4 ± 8.7 µm (film B-loaded and film B respectively). As expected, the introduction of PYC in the polymeric network increases the thickness of both films.

**Figure 6:**
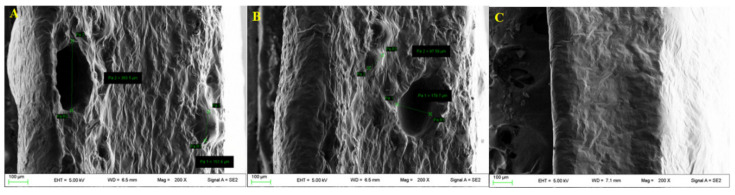
Micrographs of the sections of film A (**A**,**B**) and film B (**C**); (*n* = 3).

Moreover SEM analyses of sections showed the presence of both large pores (Figure 6A, diameter 264 ± 93 µm) and small pores (Figure 6B, diameter 147 ± 34 mm), for film A while no presence of pores for film B (Figure 6C) was detected.

### 3.9. Thermogravimetric Analysis

**Figure 7:**
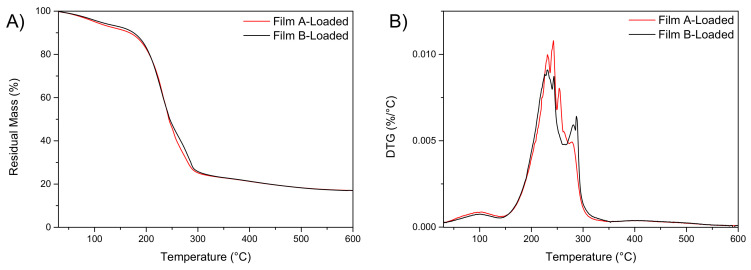
TGA (**A**) and DTG (**B**) curves of film A-loaded and film B-loaded (*n* = 3).

The effect of PYC introduction on the thermal stability of loaded films (film A-loaded and film B-loaded) was studied by TGA (Figure 7). The obtained results showed that, in both cases, the water content is below 5% at 100 °C (Figure 7A). Figure 7B shows the derivative curves (DTG) of neat films A-loaded and B-loaded, characterized by the presence of a multi-step degradation behaviour. A comparison of the weight loss curves for PYC loaded and not loaded shows that higher residual weight can be found for the films containing PYC (estimated at 20%) while the residual mass for film A and film B was measured as 10% [30]. Considering the water content, the exact film compositions were calculated (Table 4).

### 3.10. Mechanical Characterization

**Figure 8:**
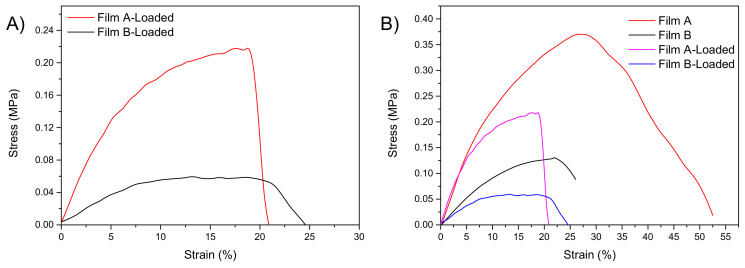
Stress-strain curves of loaded films (**A**) and loaded and unloaded film A and B (**B**) (*n* = 5).

To evaluate how PYC introduction in the composition modifies the mechanical performance of resulting films, the tensile behaviors of film A-loaded and film B-loaded were studied. The stress-strain curves of loaded films with PYC are represented in Figure 8A, while the data of film A-loaded vs film B-loaded are reported in Table 5. Similarly to what observed for the unloaded films, the best results were obtained for film A (Figure 8A,B). In fact, the higher value for the elastic modulus (E) was obtained for film A, suggesting that PYC introduction in the polymeric composition does not modify the deformability properties of the formulation. Figure 8B shows the stress strain curves of the produced films. The presence of PYC limits the mechanical performance of both film A and film B (Figure 8B). As reported in literature, the introduction of active ingredients in polymeric matrices generally modifies the tensile parameters [39,40]. The analysis of different systems confirmed that higher values for elastic modulus and tensile strength can be obtained, both in the case of unloaded and PYC loaded samples, for formulations based on film A. For this reason, as film A-loaded met the fixed requirements in terms of mechanical properties, this formulation was chosen and deeply characterized.

### 3.11. Ex Vivo Adhesion Capacity

The adhesion force and capacity of film A-loaded was measured as well. The obtained results showed that the bioadhesion force of film A after loading (0.25 ± 0.13 N) as well as the adhesion time (17.33 ± 4.50 sec) are similar to the corresponding unloaded ones (0.20 ± 0.17 N, 14.66 ± 5.68 sec), suggesting that PYC introduction does not modify this property. The adhesion capacity is attributable both to XG presence and to the morphology of this formulation. In fact, the wrinkled surface, as observed in the micrographs (Figure 5A,B), increases the surface area, enhancing the contact area between film and skin and so the bioadhesion.

### 3.12. Release Studies

**Figure 9:**
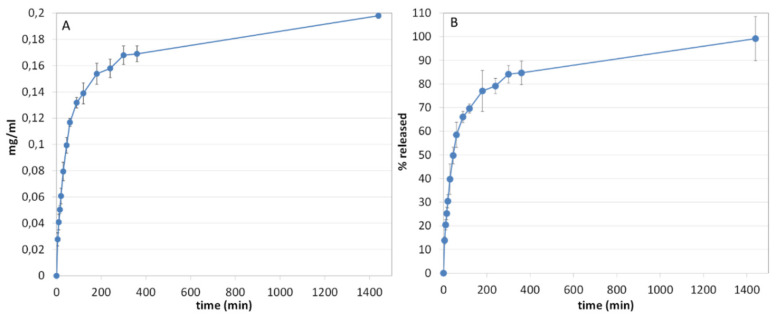
In vitro release profiles obtained from plotting concentration (**A**) vs film A-loaded time and % released (**B**) vs time (*n* = 3).

The release capacity of PYC from film A-loaded was evaluated using the in vitro method for transdermal films, according to Ph. Eur. 10^th^ Ed. Results showed that PYC is almost completely released within 24 h (Figure 9). As shown in Figure 9A (µg/mL vs time), a sustained release of PYC was obtained reaching ~ 13% of PYC release after 5 min, ~ 39% after 30 min and ~58% after 60 min (Figure 9B). To understand the kinetics responsible for PYC release from the film, the in vitro release (% released vs time) data were processed by the following mathematical models: zero-order, first-order and Higuchi. Zero- and first-order models can be applied when the release rate is, respectively, not dependent and dependent on the concentration of the active ingredient. The Higuchi model instead fits a time-dependent release based on Fickian diffusion. In this case, the best fitting was observed for the Higuchi model (Table 6), suggesting that PYC is mainly released by a diffusion-based mechanism.

### 3.13. Antimicrobial Activity Assay

It has been shown that a formulation containing 0.025% PYC shows bacteriostatic activity against gram-positive and gram-negative strains [10]. Two different water solutions of PYC (1 mg/mL and 10 mg/mL) were assessed for antimicrobial activity (Table S1). Results summarized in Table 7 show a concentration of 10 mg/mL is effective against *S. aureus*, *S. epidermidis*, *E. faecalis*, *B. subtilis*, *S. pyogenes* (Figure S2). Except for *S. aureus*, such antimicrobial activity had not been previously reported in literature [10]. As observed in other natural compounds [41], the phenolic compounds present in PYC are the ones responsible for inhibiting bacterial cell growth.

Interestingly, the strains resulted sensitive to PYC are often involved in wound infections [42,43,44]. The antimicrobial activity of PYC loaded in film A was evaluated as well. In fact, the study of PYC antimicrobial activity in the final formulation is very important as PYC is released from the polymeric matrix by a sustained mechanism. Results (Table 7) show that film A-loaded can inhibit growth in three strains *E. faecalis*, *S. pyogenes* and *S. aureus* (Figure S3). The unloaded film (PYC free) was assayed as control and no inhibition was observed. The inhibition halo observed both for *E. faecalis* and *S. aureus* is comparable to that obtained from the solution, while in the case of *S. pyogenes* it increases; no effect on *S. epidermidis* and *B. subtilis* was instead observed.

### 3.14. Cytotoxicity and Wound Healing

To evaluate the safety of PYC concentrations obtained from film-loaded A release, an in vitro cytotoxicity study (MTT test) was performed. Human immortalized keratinocytes (HaCaT) were used as model cell system representative of stratum corneum. Cells were incubated with different dilutions of the stock PYC solution (1.48 mg).

**Figure 10:**
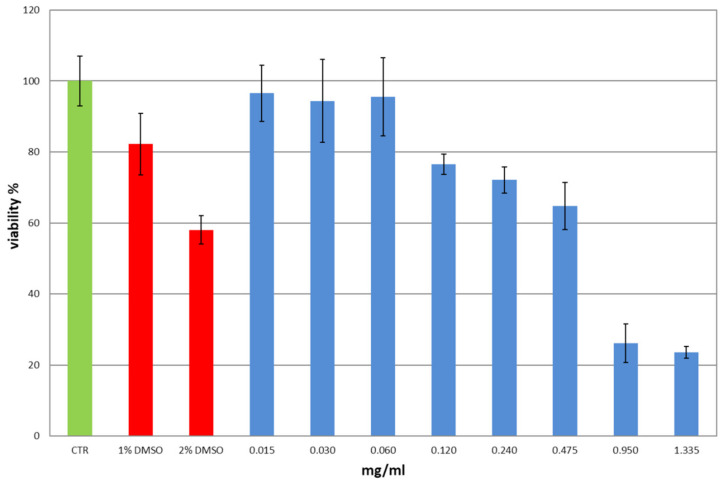
Viability measured in vitro on HaCaT cells for different PYC concentrations (0.015–1.335 mg/mL). CTR, untreated cells in DMEM were set at 100%. DMSO in three different percentages (1%, 2% and 4%) as positive controls (*n* = 3).

The obtained results (Figure 10) show that PYC is cytotoxic in dose-dependent manner at the highest concentrations assayed, namely 0.95 and 1.335 mg/mL (viability < 30%). Except for the concentration 0.120–0.475 mg/mL (viability > 60%), in all the other cases the measured viability was ≥ 70%, supporting the safety of PYC. In the case of the lowest concentrations, 0.015–0.060 mg/mL (viability > 95%), the standard deviation cells viability, is comparable to the control cell (CTR). The empty film was also assayed as a further control experiment; no cytotoxicity was observed (Figure S4). The concentrations observed in the in vitro release studies (Figure 9A) are within the safety levels (Figure 10).

Based on both MTT results and release data, it was decided to perform the scratch test using the lowest concentrations: 0.015 and 0.03 mg/mL for which cell viability is approximately around 100% (Figure 10).

**Figure 11:**
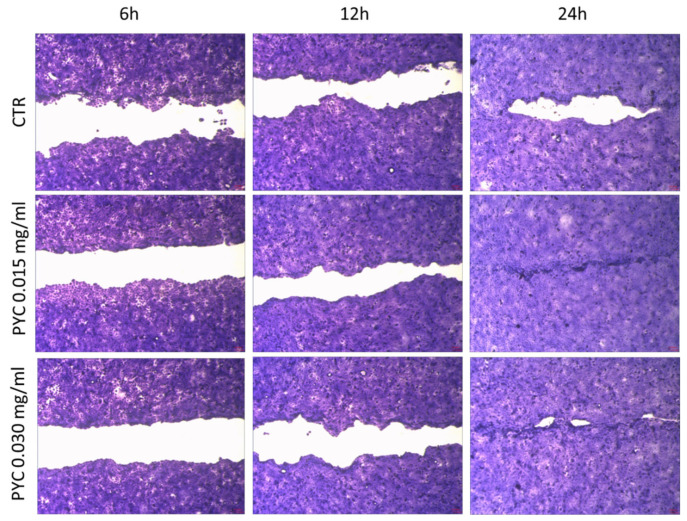
Pictures of scratch test performed on untreated HaCat cells (CTR) and treated with two different PYC concentrations 0.015 mg/mL and 0.030 mg/mL (*n* = 2).

For the wound healing test, the inserts were removed when the human keratinocyte reached approximatively 80% confluence. The total wound field surface area is 1.62 mm^2^. Untreated cells show a 54.7% ± 5.1%, 72.1% ± 7.8%, 93.2% ± 4.9 closure after 6, 12 and 24 h, respectively. As shown in Figure 11, PYC can stimulate cell growth; interestingly, results showed differences between treated and untreated cell.

The best result is achieved after 24 h of treatment with the lowest PYC concentration tested (0.015 mg/mL). In fact, the wound is completely closed (100% of closing). Interesting differences between the two assessed concentrations, in comparison to control cells (CTR), are obtained after 6 and 12 h of treatment. Compared to the CTR, the ability to stimulate cell growth can be observed after both 6 and 12 h of treatment. Within 6 h, PYC 0.015 mg/mL and PYC 0.03 mg/mL exhibit percent closures equal to 62.5% ± 2.6%, and 57.9 ± 1.8%, respectively; the first one than the one measured for the CTR (54.7% ± 5.1%). It is interesting to note that after 12 h, cells treated with the 0.015 mg/mL PYC solution show a decreased area of the wound field (88.9 ± 3.2% of closing). Instead, such enhanced healing activity was not found in the cells treated with the higher PYC concentration (0.030 mg/mL), which achieves only 75.1% ± 4.4% (comparable to CTR 72.1% ± 7.8%). At the 24-h end-point, wound closure nears 100% for both PYC concentrations (Figure 11) vs CTR in which the wound field is still open.

## 4. Conclusions

Picnogenol (PYC) is a viable molecule for wound treatment. It was formulated in bioadhesive films obtained from a mixture of the biopolymers xanthan gum and alginic acid sodium salt hydrogels. The film showed suitable mechanical properties such as high deformability, suggesting easy adaptability to any type of surface. The film composition was found to be capable of easy adhesion to skin and of absorbing the exudates from the wound. In vitro assays demonstrated that the developed films are active against the *S. pyogenes*, *S. aureus* and *E. faecalis* bacterial strains. The sustained release of PYC from the formulation suggests that this formulation could be applied once-per-day, allowing a complete protection of the damaged area and promoting the healing also by stimulating keratinocytes growth.

## Figures and Tables

**Table 1:** Compositions of the hydrogel mixtures (AL/XG) chosen.

Hydrogel	AL (% wt/wt)	XG (% wt/wt)	Glycerol (% wt/wt)	Bidistilled Water (% wt/wt)
A	2.50	7.50	10.00	80.00
B	1.50	8.50	10.00	88.00

**Table 2:** Films water loss obtained after one day of storage at different conditions.

Film	Storage Conditions	Water Loss (%)
A	ventilated oven at 42 °C	4.96 ± 1.58
	desiccator under CaCl_2_	6.51 ± 1.58
	desiccator under P_2_O_5_	6.10 ± 3.35
B	ventilated oven at 42 °C	4.33 ± 1.01
	desiccator under CaCl_2_	9.77 ± 3.76
	desiccator under P_2_O_5_	8.30 ± 1.25

**Table 3:** Stress at break (σ_max_), deformation at break (ε_at σmax_) and elastic modulus (E) measured for film A and film B; * *p* < 0.001; ** *p* > 0.05; *** *p* < 0.001 film A vs film B.

Film	AL/XG (Ratio wt/wt)	σ_max_ (MPa)	ε_at σmax_ (%)	E (MPa)
A	2.5/7.5	0.303 ± 0.077 *	23 ± 4 **	2.823 ± 0.148 ***
B	1.5/8.5	0.120 ± 0.010	22 ± 4	1.278 ± 0.169

**Table 4:** Film A-Loaded and film B-Loaded compositions.

Film	AL (% wt/wt)	XG (% wt/wt)	PYC (% wt/wt)	Glycerol (% wt/wt)	Water (% wt/wt)
A-Loaded	1.50	3.02	20.10	40.20	35.17
B-Loaded	0.87	3.40	20.30	40.61	34.81

**Table 5:** Mechanical parameters measured for loaded films; * *p* < 0.001; ** *p* > 0.05; *** *p* < 0.001 film A vs film B.

	*σ_max_ (MPa)*	*ε_at σmax_ (%)*	*E(MPa)*
**Film A-Loaded**	0.215 ± 0.007 *	17 ± 2 **	3.070 ± 0.044 ***
**Film B-Loaded**	0.055 ± 0.005	18 ± 1	0.620 ± 0.044

**Table 6:** Mathematical models of in vitro release data.

	M_t_/M_∞_ = kt	M_t_/M_∞_ = kt^0.5^	M_t_/M_∞_ = 1-e^-kt^
	**Zero-order Kinetics**	**Higuchi Kinetics** **(Release 0–60%)**	**First Order Kinetics**
**Film A-loaded**	y = 0.0447x + 46.761 R^2^ = 0.3877	y = 6.1205x + 0.1606 R^2^ = 0.9869	y = −0.0007x − 0.3057 R^2^ = 0.6355

**Table 7:** Inhibition halos measured for PYC solutions and film A-loaded (*n* = 3).

	PYC 10 mg/mL (mm)	PYC 1 mg/mL (mm)	film A-Loaded (mm)
*K. pneumoniae*	-	-	-
*E. coli*	-	-	-
*P. mirabilis*	-	-	-
*S. aureus*	19	-	19
*S. epidermidis*	20	-	-
*E. faecalis*	17	-	18
*B. subtilis*	17	-	-
*S. pyogenes*	21	-	24
*P. aeruginosa*	-	-	-
*C. albicans*	-	-	-

## Data Availability

Not applicable.

## Supplementary Materials

The following are available online at https://www.mdpi.com/1999-4923/13/3/324/s1, Figure S1: title, Table S1: title, Video S1: title.Growth conditions of the strains used for the antimicrobial activity assay; Table S2. Different conditions assayed for film storage and observations after 7 days; Figure S1. TGA profiles of AL, XG and glycerol (a); DTG profiles of AL, XG and glycerol (b); Figure S2. (a) *S. aureus*; (b) *S. epidermidis*; (c) *E. faecalis*; (d) *B. subtilis*; (e) *S. pyogenes*; Figure S3. Film A-loaded: (a) *E. faecalis*, (b) *S. pyogenes*, (c) *S. aureus;* Figure S4. Viability measured in vitro on HaCaT cells incubated with different volumes of DMEM previously incubated for 24 h with the patch (2 × 2 cm in 10 mL of DMEM) free from PYC. CTR, untreated cells in DMEM were set at 100%. DMSO in three different percentages (1%, 2% and 4%) as positive controls (*n* = 3).
