# Supplementary material for: Development and Characterization of Xanthan Gum and Alginate Based Bioadhesive Film for Pycnogenol Topical Use in Wound Treatment
Source: Pharmaceutics. 2021 Mar 3;13(3):324. doi: 10.3390/pharmaceutics13030324 (PMC8002000; doi:10.3390/pharmaceutics13030324)
Supplement: Supplementary file 1 [file pharmaceutics-13-00324-s001.pdf]

pharmaceutics

MDPI

Supplementary Materials: Development and Characterization of Xanthan Gum and Alginate Based Bioadhesive Film for Pycnogenol Topical Use in Wound Treatment

Cinzia Pagano, Debora Puglia, Francesca Luzi, Alessandro Di Michele, Stefania Scuota, Sara Primavilla, Maria Rachele Ceccarini, Tommaso Beccari, César Antonio Viseras Iborra, Daniele Ramella, Maurizio Ricci and Luana Perioli

Table S1. Growth conditions of the strains used for the antimicrobial activity assay.

Gram positive bacteria	Growth conditions
Staphylococcus epidermidis WDCM 00036	37°C for 24 ± 2 hours
Enterococcus faecalis WDCM 00087	37°C for 24 ± 2 hours
Bacillus subtilis WDCM 00003	30°C for 24 ± 2 hours
Staphylococcus aureus WDCM 00034	37°C for 24 ± 2 hours
Streptococcus pyogenes ATCC 19615	37°C for 24-48 hours
**Gram negative bacteria**	
Pseudomonas aeruginosa WDCM 00025	25°C for 24-48 hours
Klebsiella pneumoniae WDCM 00097	37°C for 24 ± 2 hours
Proteus mirabilis WDCM 00023	37°C for 24 ± 2 hours
Escherichia coli WDCM 00013	37°C for 24 ± 2 hours
**Yeast**	
Candida albicans WDCM 00054	25°C for 24-72 hours

Publisher's Note: MDPI stays neutral with regard to jurisdictional claims in published maps and institutional affiliations.

Copyright: © 2021 by the authors. Submitted for possible open access publication under the terms and conditions of the Creative Commons Attribution (CC BY) license (http://creativecommons.org/licenses/by/4.0/).

Table S2. Different conditions assayed for film storage and observations after 7 days.

Film	AL/XG hydrogel ratio (wt./wt.)	storage conditions	observations after 7 days
**A**	2.5/7.5	$CaCl_2$ at R.T.	the initial characteristics are maintained
**B**	1.5/8.5	$CaCl_2$ at R.T.	the initial characteristics are maintained
**A**	2.5/7.5	saturated $MgCl_2$ solution at 4°C	water absorption makes the film gelatinous
**B**	1.5/8.5	saturated $MgCl_2$ solution at 4°C	water absorption makes the film gelatinous (more than A)
**A**	2.5/7.5	saturated $MgCl_2$ solution at R.T.	the initial characteristics are maintained
**B**	1.5/8.5	saturated $MgCl_2$ solution at R.T.	the initial characteristics are maintained

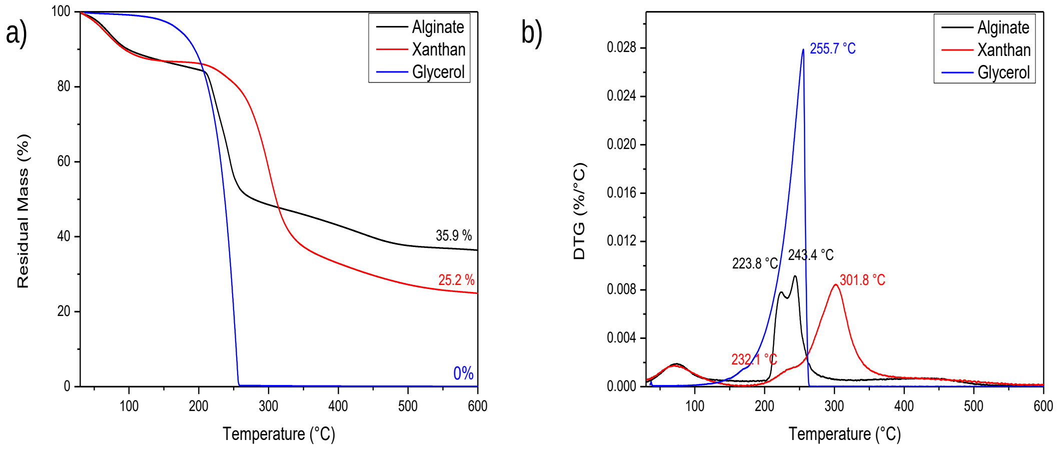

Figure S1. TGA profiles of AL, XG and glycerol (a); DTG profiles of AL, XG and glycerol (b).

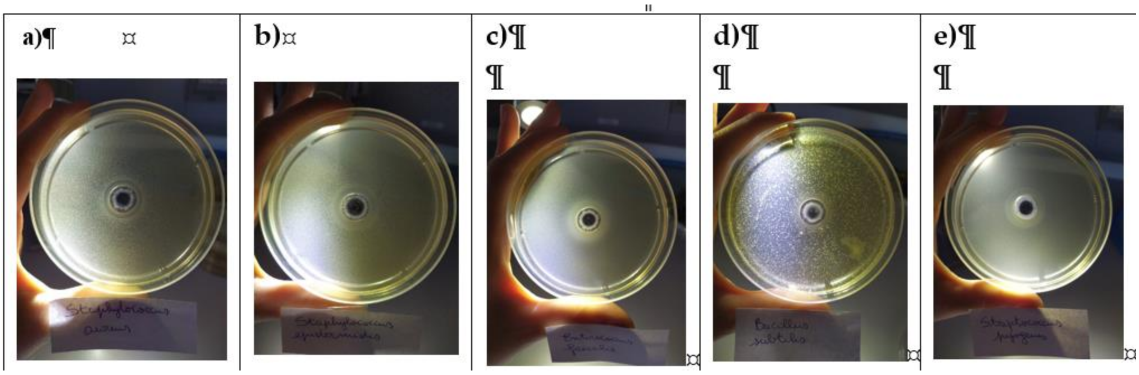

Figure S2. a) S. aureus; b) S. epidermidis; c) E. faecalis; d) B. subtilis; e) S. pyogenes.

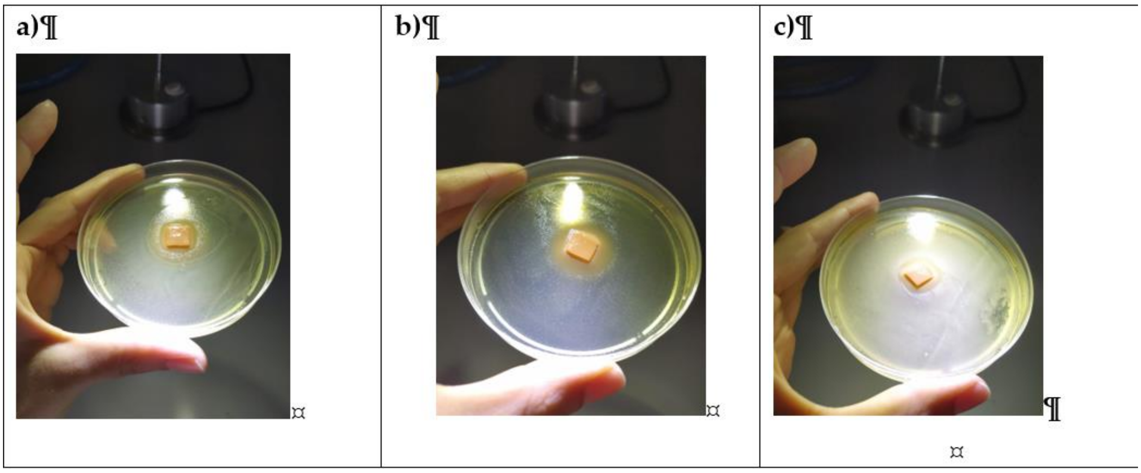

Figure S3 Film A-loaded: a) *E. faecalis*, b) *S. pyogenes*, c) *S. aureus*.

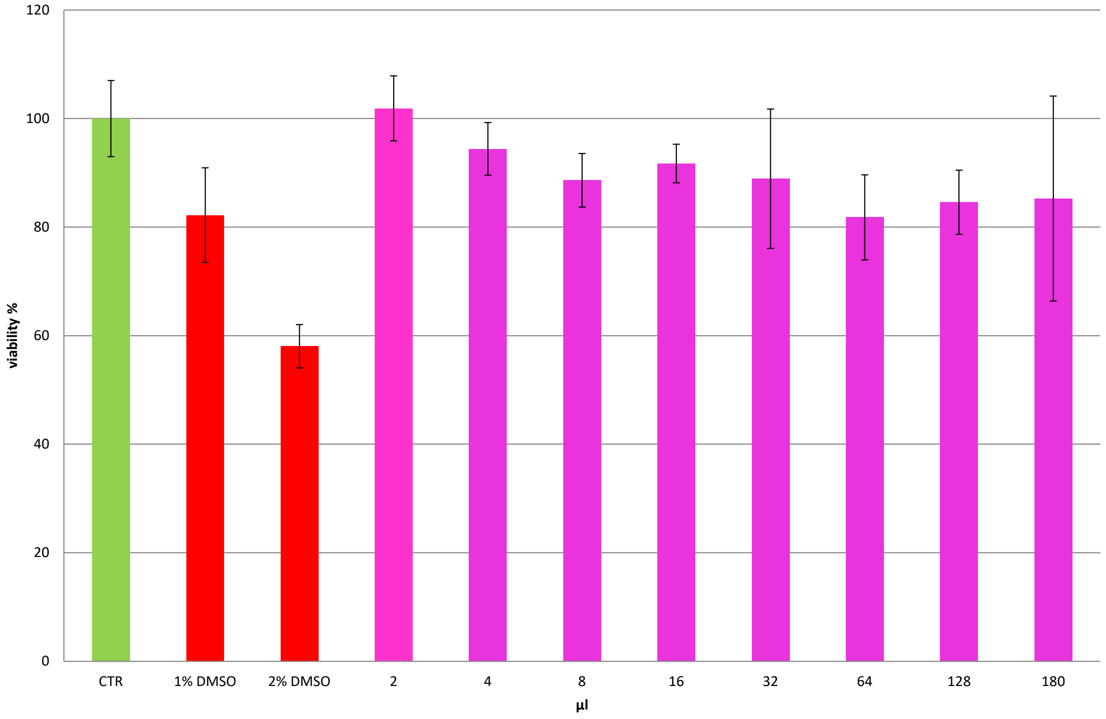

Figure S4. Viability measured in vitro on HaCaT cells incubated with different volumes of DMEM previously incubated for 24 h with the patch (2 x 2 cm in 10 ml of DMEM) free from PYC. CTR, untreated cells in DMEM were set at 100%. DMSO in three different percentages (1%, 2% and 4%) as positive controls (n = 3).
